# Phytoconstituents of *Withania somnifera* unveiled Ashwagandhanolide as a potential drug targeting breast cancer: Investigations through computational, molecular docking and conceptual DFT studies

**DOI:** 10.1371/journal.pone.0275432

**Published:** 2022-10-06

**Authors:** Hittanahallikoppal Gajendramurthy Gowtham, Mahadevamurthy Murali, Sudarshana Brijesh Singh, Chandan Shivamallu, Sushma Pradeep, C. S. Shivakumar, Satish Anandan, Anjana Thampy, Raghu Ram Achar, Ekaterina Silina, Victor Stupin, Joaquín Ortega-Castro, Juan Frau, Norma Flores-Holguín, Kestur Nagaraj Amruthesh, Shiva Prasad Kollur, Daniel Glossman-Mitnik

**Affiliations:** 1 Department of Biotechnology, Government Science College Autonomous, Bengaluru, Karnataka, India; 2 Applied Plant Pathology Laboratory, Department of Studies in Botany, University of Mysore, Manasagangotri, Mysore, Karnataka, India; 3 Department of Studies in Biotechnology, University of Mysore, Mysore, Karnataka, India; 4 Department of Biotechnology and Bioinformatics, School of Life Sciences, JSS Academy of Higher Education and Research, Mysuru, Karnataka, India; 5 Department of Clinical Nutrition and Dietetics, Sri Devaraj Urs Academy of Higher Education and Research, Kolar, Karnataka, India; 6 Division of Biochemistry, School of Life Sciences, JSS Academy of Higher Education and Research, Mysuru, Karnataka, India; 7 Department of Human Pathology, I. M. Sechenov First Moscow State Medical University (Sechenov University), Moscow, Russia; 8 Department of Hospital Surgery, N.I. Pirogov Russian National Research Medical University (RNRMU), Moscow, Russia; 9 Departament de Química, Universitat de les Illes Balears, Palma de Malllorca, Spain; 10 Laboratorio Virtual NANOCOSMOS, Departamento de Medio Ambiente y Energía, Centro de Investigación en Materiales Avanzados, Chih, México; 11 School of Agriculture, Geography, Environment, Ocean and Natural Sciences (SAGEONS), University of the South Pacific, Laucala Campus, Suva, Fiji; 12 Department of Sciences, Amrita School of Arts and Sciences, Amrita Vishwa Vidyapeetham, Mysuru Campus, Mysore, Karnataka, India; Babasaheb Bhimrao Ambedkar University (A Central University), INDIA

## Abstract

Breast cancer is the second most common malignancy in females worldwide and poses a great challenge that necessitates the identification of novel therapeutic agents from several sources. This research aimed to study the molecular docking and molecular dynamics simulations of four proteins (such as PDB: 6CBZ, 1FDW, 5GWK and 2WTT) with the selected phytochemicals from *Withania somnifera* to identify the potential inhibitors for breast cancer. The molecular docking result showed that among 44 compounds, two of them, Ashwagandhanolide and Withanolide sulfoxide have the potential to inhibit estrogen receptor alpha (ERα), 17-beta-hydroxysteroid -dehydrogenase type 1 (17β-HSD1), topoisomerase II alpha (TOP2A) and p73 tetramerization domain that are expressed during breast cancer. The molecular dynamics (MD) simulations results suggested that Ashwagandhanolide remained inside the binding cavity of four targeted proteins and contributed favorably towards forming a stable protein-ligand complex throughout the simulation. Absorption, Distribution, Metabolism, Excretion and Toxicity (ADMET) properties confirmed that Ashwagandhanolide is hydrophobic and has moderate intestinal permeability, good intestinal absorption, and poor skin permeability. The compound has a relatively low VDss value (-1.652) and can be transported across ABC transporter and good central nervous system (CNS) permeability but did not easily cross the blood-brain barrier (BBB). This compound does not possess any mutagenicity, hepatotoxicity and skin sensitization. Based on the results obtained, the present study highlights the anticancer potential of Ashwagandhanolide, a compound from *W. somnifera*. Furthermore, *in vitro* and *in vivo* studies are necessary to perform before clinical trials to prove the potentiality of Ashwagandhanolide.

## Introduction

Cancer is the leading cause of death worldwide, with a projected 19.3 million new cases reported and approximately 10 million deaths in 2020. It has been estimated that about 26 million new cases and 17 million deaths by 2030 [1, 2]. Cancer develops when normal cells are transformed into cancer cells that usually evolve in a multi-stage and multi-step process from the pre-cancerous lesions to malignant (cancerous) tumors. These changes result from a person’s hereditary factors interacting with external agents (including physical, chemical and biological carcinogens). Breast cancer is the most common cancer among women globally and the second greatest cause of death after lung cancer. It is the most common women diagnosed cancer with an estimated 2.3 million new cases and nearly 685,000 deaths per year globally in 2020 [3]. Surgery, radiation, hormone therapy, chemotherapy, and targeted biological therapy are the most common treatments for breast cancer. However, scientists are still focusing on novel therapies and medications and innovative combinations of existing treatments. New anticancer drugs or other chemicals are used in targeted therapy to attack cancer cells with less harm to normal cells. Since cancer is a complex and multi-gene disease, promising research is presently focused on developing multi-targeted safe potent anticancer drugs to improve therapeutic efficacy and prevent drug resistance. *Withania somnifera* (Ashwagandha), an ayurvedic plant from the Solanaceae family, has been used in India for more than 3000 years for its many health benefits, including stress management, increasing energy levels and cognitive health, and reducing the level of blood sugar, inflammation, anxiety, depression and cortisol [4]. It has been well documented from the literature that the plant *W. somnifera* is found throughout the world in xeric and drier of tropical and subtropical areas [5]. This important medicinal plant and its leave, root, stem and flower extracts have been discovered to have anti-microbial, anti-oxidant, anti-diabetic, anti-inflammatory, anticancer, anti-epileptic, anti-arthritic, anti-depressant, anti-coagulant, anti-pyretic properties as well as palliative effects like analgesic, regeneration, rejuvenation, and health-promoting potential [6]. Despite its long history of medical usage in many different places of the world, the basic mechanistic research on the potential of *W. somnifera* extracts has only lately been investigated in clinical trials. As a result, several more randomized double-blind placebo control studies utilizing *W. somnifera* in interventional clinical trials have found that *W. somnifera* was not only effective at a specified dosage ranging from 200–1000 mg/kg, but most notably it was safe and well-tolerated at these dosages [7].

For the first time, Chakraborti et al [8] showed that the changes in the anticancer components of *W. somnifera* and *in vivo* potential growth inhibitory activities of the plant’s root extracts in the transplantable mouse Sarcoma 180 tumor. In this contrast, the involvement of *W. somnifera* in cancer prevention was discovered in 1992, and it was found to inhibit the formation of new cancer cells but not normal cells, aid to induce programmed cell death by producing reactive oxygen species (ROS), and finally to sensitize the cancer cells to apoptosis [9–11]. The active constituents and their effects in cancer prevention have been studied using several types of *W. somnifera* extracts of different parts (such as leaf, stem, root and fruit) of the plant as well as the isolated withanolides have been explored as potential anti-carcinogenic agents in the brain, breast, cervical, prostate, lung, colon and other cancers [7].

Previously, the researchers have reported that the edaphic factors had no significant correlation in the phytoconstituents of the plant. In this context, the analysis of genetic variability in natural populations performed by many researchers has noted that the plant is found to be polymorphic in nature at their phenotypic and chemotypic levels and the variations in the phytoconstituents are largely genetic in nature [12–15].

Recent studies have demonstrated that some bioactive molecules (such as Withaferin A) of *W. somnifera* can arrest breast cancer development by targeting ERα using *in silico* computational approaches [16, 17]. However, other phytochemicals isolated from *W. somnifera* have not yet been checked for inhibiting the multiple proteins responsible for breast cancer by utilizing the *in silico* approaches. With this background, the molecular docking and molecular dynamics (MD) simulation were achieved in the present study to explore the potential of the phytochemicals from *W. somnifera* as a promising drug candidate against four proteins expressed during breast cancer that could be developed and used as drugs for the treatment of cancer. Additionally, ADMET prediction analysis was conducted to determine further the potential compounds’ drug-likeness, pharmacokinetics, and pharmacodynamic parameters. This was complemented with a computational prediction of the Conceptual DFT descriptors of chemical reactivity of the Ashwagandhanolide phytochemical.

## Materials and methods

### Ligands optimization

In the present study, a total of 44 phytochemicals from *Withania somnifera* were selected as the ligand molecules [18]. The selected compounds’ 3D and 2D structures (SDF files) were downloaded from the online PubChem database (https://pubchem.ncbi.nlm.nih.gov/). Some of the compounds were drawn using Marvinsketch Chemaxon chemical drawing tool (https://chemaxon.com/products/marvin/). The 2D structure was converted into 3D coordinates and geometries and converted into PDB files using Open Babel [19].

### Proteins preparation

The four protein receptors used [2] are estrogen receptor alpha (ERα) ligand-binding domain Y537S mutant in complex with estradiol and GRIP peptide (PDB: 6CBZ, Resolution: 1.65 Å), human 17-beta-hydroxysteroid-dehydrogenase type 1 (17β-HSD1) mutant H221Q complexed with estradiol (PDB: 1FDW, Resolution: 2.70 Å), human topoisomerase II alpha (TOP2A) in complex with DNA and etoposide (PDB: 5GWK, Resolution: 3.15 Å), and human p73 tetramerization domain (PDB: 2WTT, Resolution: 2.30 Å). The protein structures were downloaded from the Protein Data Bank archive-information (https://www.rcsb.org/) as PDB files and processed by removing ligand and water molecules attached to these enzymes to avoid interfering with the docking study. Discovery Studio software was used to compute energy minimization, then reconstruct missing atoms and perform stereo-chemical quality checks to arrive at the best feasible 3D structures [20].

### Validation of structure of proteins

The structure of proteins was further validated by using the Ramachandran plot, which is generated by using PROCHECK via PDBsum database (http://www.ebi.ac.uk/thornton-srv/databases/pdbsum/Generate.html) that enable visualization of highly preferred, preferred, and disallowed phi (ϕ) and psi (ψ) angles of each amino acid in the protein. The structure was also checked with the Protein Structure Analysis (ProSA) web tool to ascertain the overall model quality of the protein and generate the Z-score for a given input protein. Z-score should be within the range of native proteins for a high-quality protein model; otherwise, the protein structure may have mistakes.

### Molecular docking study

The molecular docking study was achieved to carry molecular docking calculations of the phytochemicals from *W. somnifera* with four protein receptors using AutoDock Vina implicated in PyRx. The conformation with the lowest binding energy (kcal/mol) was chosen as the best docking pose. Discovery Studio was also used to investigate the interactions between ligand and protein [21].

### Molecular Dynamics (MD) molecular simulations

The MD simulations of protein and protein-ligand complexes were performed using CHARMM27 all-atom additive force fields in the GROMACS simulation package. The SwissParam webserver was used to generate the topologies and parameter files and prepare a protein-ligand complex. To solvate, the system the simulation box, the TIP3P water model was employed, and additional NaCl counter ions and co-ions were added to neutralize the whole solvated system charge [22–26]. The structures were minimized using the steepest descent algorithm, and the system was equilibrated with canonical (NVT) ensemble and then isothermal-isobaric (NPT) ensemble during simulation. Finally, the simulation (production run) was of 50 ns equilibration run at the desired temperature (300 K) and pressure (1 bar) [27, 28].

### Prediction of Absorption, Distribution, Metabolism, Excretion and Toxicity (ADMET) properties of ligands

The ADMET physicochemical properties such as Log Po/w, molecular weight, number of carboxylic groups and number/type of substituents attached to the backbone of ligands are crucial to predict the human serum albumin (HSA) binding of drugs [29]. An integrated, freely available pkCSM platform was employed to predict ADMET properties of the selected potential ligand molecule [19]. It predicted the absorption parameters [such as water solubility in a buffer system, Caco2 cell permeability, intestinal (human) absorption, P-glycoprotein inhibition and skin permeability], metabolic parameters [such as Cytochrome P450 (CYP) 1A2, CYP2C19, CYP2C9, CYP2D6 and CYP3A4 inhibition, CYP2D6 and CYP3A4 substrate] and distribution properties [such as Lipinski’s rule, blood-brain barrier (BBB) and central nervous system (CNS) permeability]. The excretion efficacy of the proposed metabolite is one of the important parameters, including total renal clearance and renal OCT2 substrate. Due to their poorer renal clearance, many drugs are frequently failed at the clinical drug trial stages. The toxicity of compounds included the AMES test, oral rat acute and chronic toxicity, skin sensitization, hepatotoxicity, *Tetrahymena pyriformis* toxicity and Minnow toxicity were also computed.

### Conceptual DFT studies

The molecular energy, electronic density, and frontier orbital energies, chemical reactivity descriptors and of the studied Ashwagandhanolide molecular system were determined using the Kohn-Sham (KS) approach [30–33] while making use of the Conceptual DFT (CDFT) methodology [34–40]. Many different conformers of the studied compound were determined using MarvinView 17.15 from ChemAxon [http://www.chemaxon.com] through the consideration of the MMFF94 force field to perform Molecular Mechanics calculations [41–45]. This was followed by a geometry optimization and frequency calculation by means of the Density Functional Tight Binding (DFTBA) methodology [46] and a later geometry reoptimization, frequency analysis and calculation of the electronic properties and the chemical reactivity descriptors by means of the MN12SX/Def2TZVP/H2O model chemistry [47–49] on their optimized molecular structures. The charge of the molecule was taken as equal to zero while the radical anion and cation have been considered in the doublet spin state. This determination was performed with the aid of the Gaussian 16 software [46] and the SMD solvation model [50] and owing to the fact that the mentioned model chemistry has been previously proved as verifying the ‘Koopmans in DFT’ (KID) procedure [51–54], This last step was also required for the verification of the absence of imaginary frequencies as a check for the stability of the optimized structure as being a minimum in the energy landscape.

## Results and discussion

Even though drug discovery is a time-consuming and costly process, new drugs must meet the world’s current unmet clinical needs. As a result, the computer-aided drug discovery approaches (such as *in silico* virtual screening approach), which are faster and less expensive than *in vitro* high-throughput screening, play a vital role in dealing with such a vast number of compounds and assisting in the drug discovery process [55]. In the present study, we employed the in *silico* molecular docking analysis and molecular dynamics simulation and ADMET properties of ligands to identify the potential novel inhibitors from *W. somnifera* against the multiple target proteins of breast cancer as ERα, 17β-HSD1, TOP2A and p73 tetramerization domain.

### Validation of the structure of the proteins

**Fig 1:**
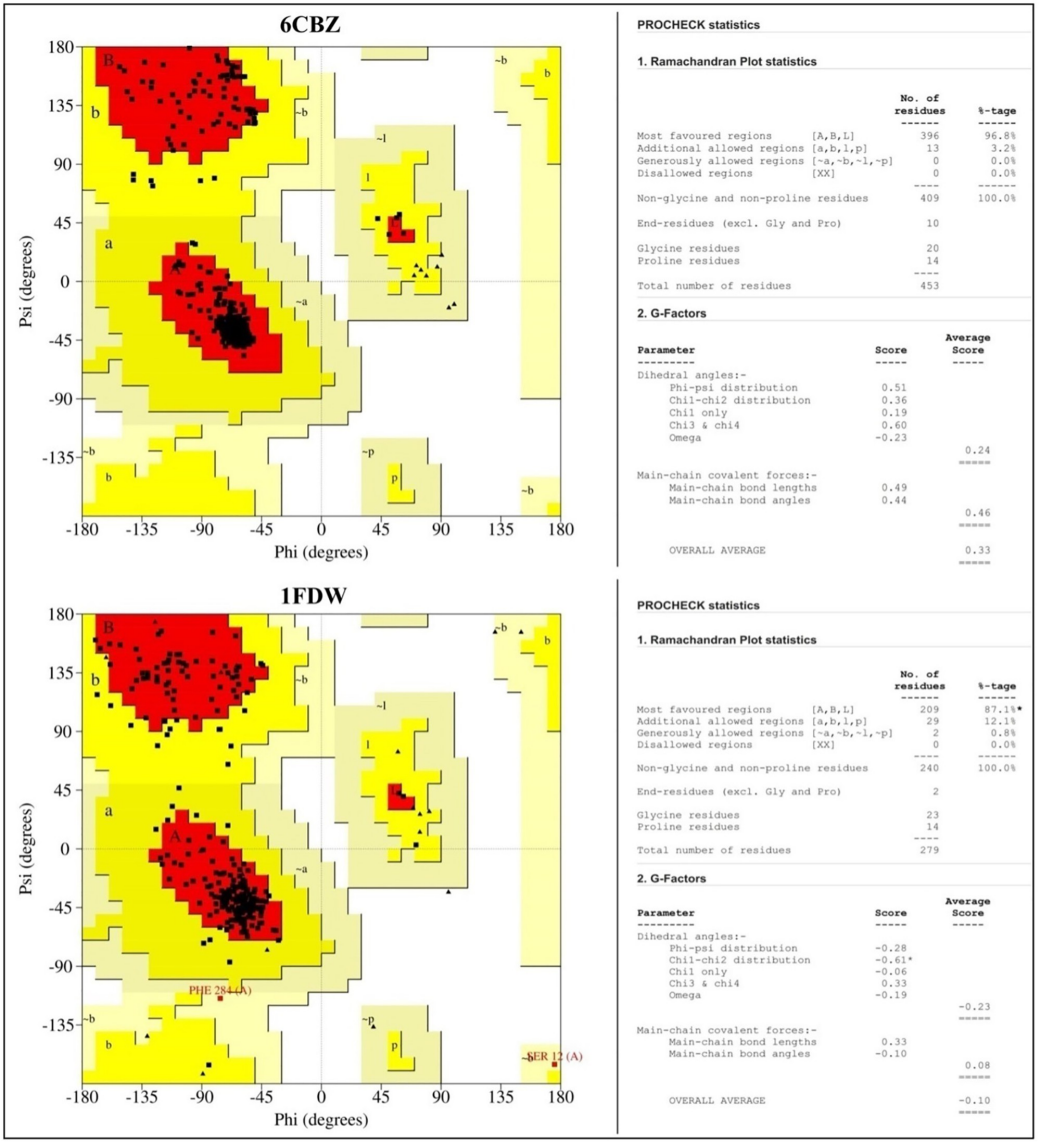
Ramachandran plot for the model of the structure of ERα (PDB: 6CBZ) and human 17β-HSD1 (PDB: 1FDW) proteins generated by PROCHECK. The red color region denotes residues of the protein in the most favored regions; the brown color denotes residues in the additional allowed regions and the yellow indicates residues in the generously allowed regions.

**Fig 2:**
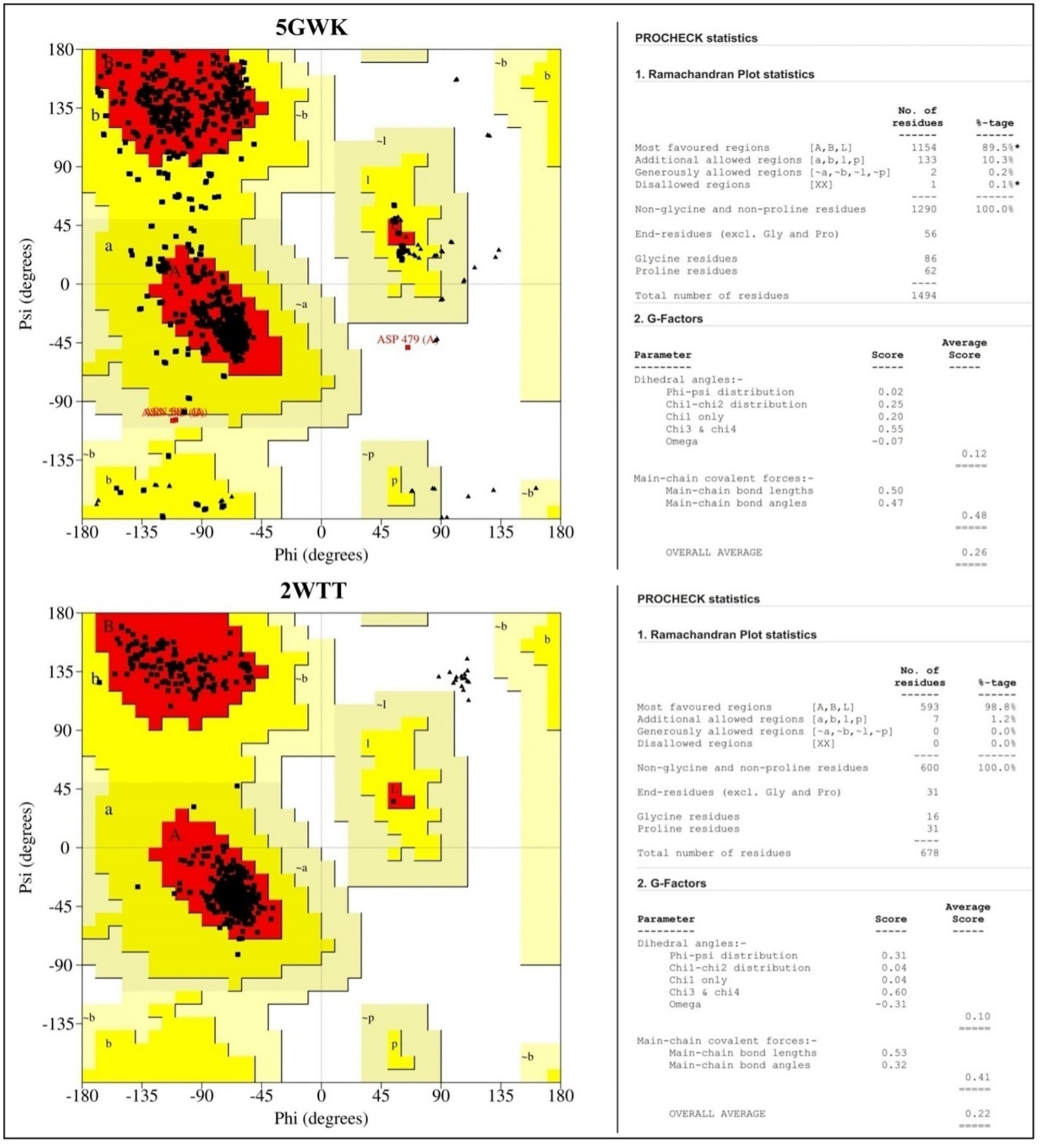
Ramachandran plot for the model of the structure of human TOP2A (PDB: 5GWK) and human p73 tetramerization domain (PDB: 2WTT) proteins generated by PROCHECK. The red color region denotes residues of the protein in the most favored regions; the brown color denotes residues in the additional allowed regions and the yellow indicates residues in the generously allowed regions.

**Fig 3:**
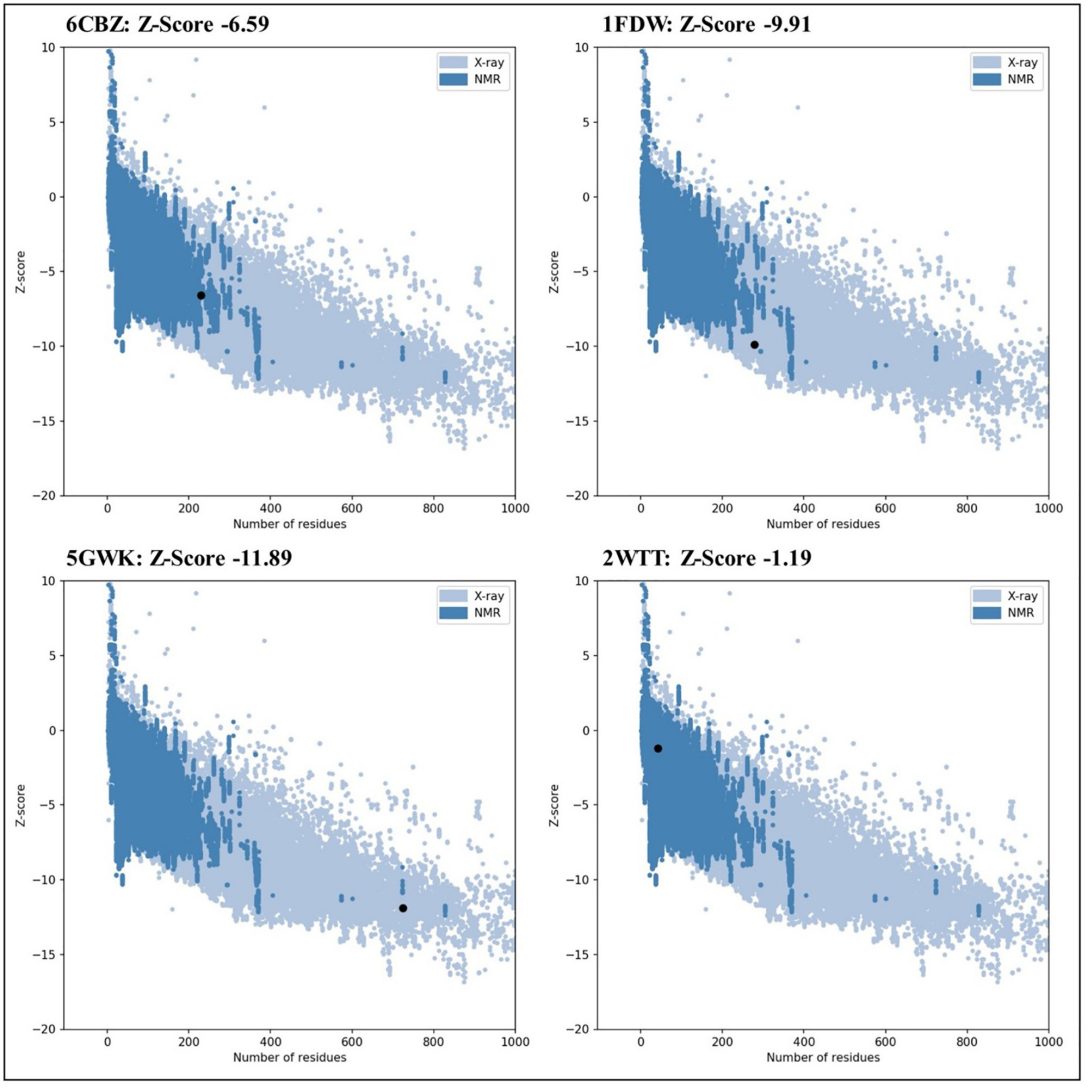
Z-score for the model of the structure of multiple proteins generated by ProSA web tool.

The obtained PROCHECK plot analysis represented that the majority of the amino acids residues are found within the most favored regions of the proteins used (Figs 1 and 2). Z-scores generated by the ProSA web tool was found within the range of their native proteins, which confirms the models of proteins used are high quality (Fig 3).

### Molecular docking analysis

**Fig 4:**
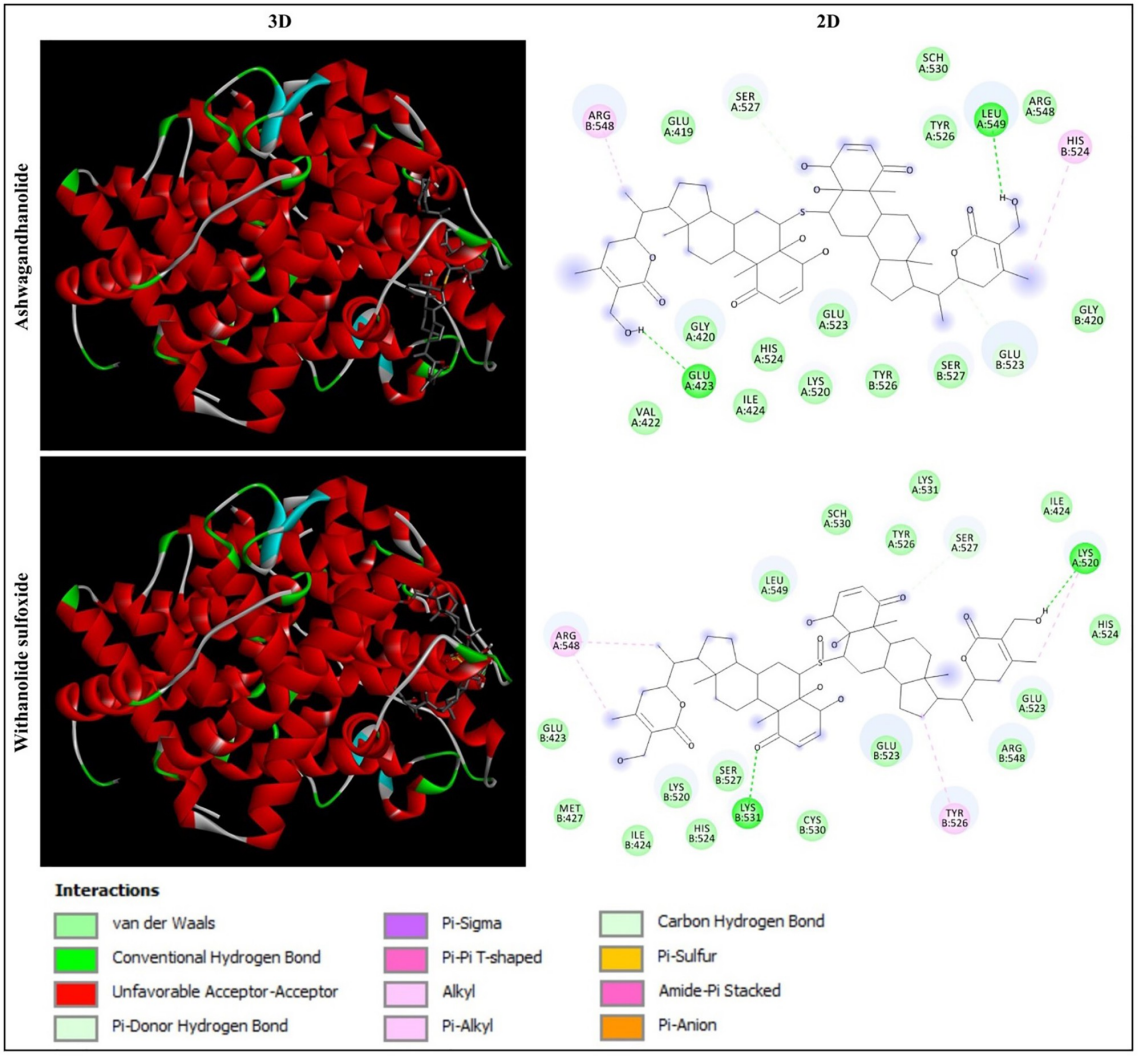
Interaction of ERα (PDB: 6CBZ) with Ashwagandhanolide and Withanolide sulfoxide. Three-dimensional (3D) illustration shows the interaction of ligands with ERα structure and two-dimensional (2D) diagram displays the interactions of the ligand with the specific amino acid residues in the active site of the protein.

**Fig 5:**
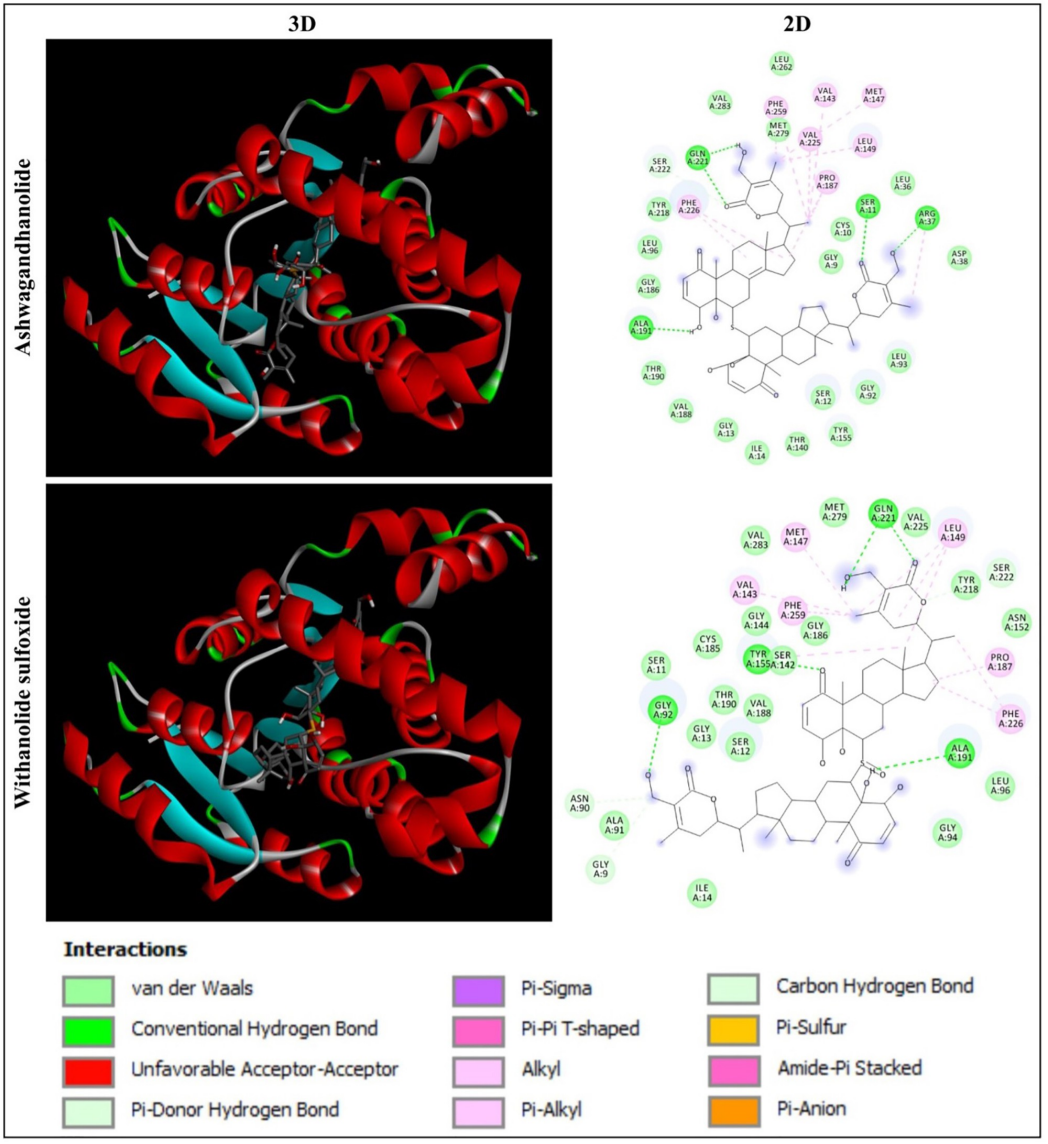
Interaction of human 17β-HSD1 (PDB: 1FDW) with Ashwagandhanolide and Withanolide Sulfoxide. Three-dimensional (3D) illustration shows the interaction of ligands with human 17β-HSD1 structure and two-dimensional (2D) diagram displays the interactions of the ligand with the specific amino acid residues in the active site of the protein.

**Fig 6:**
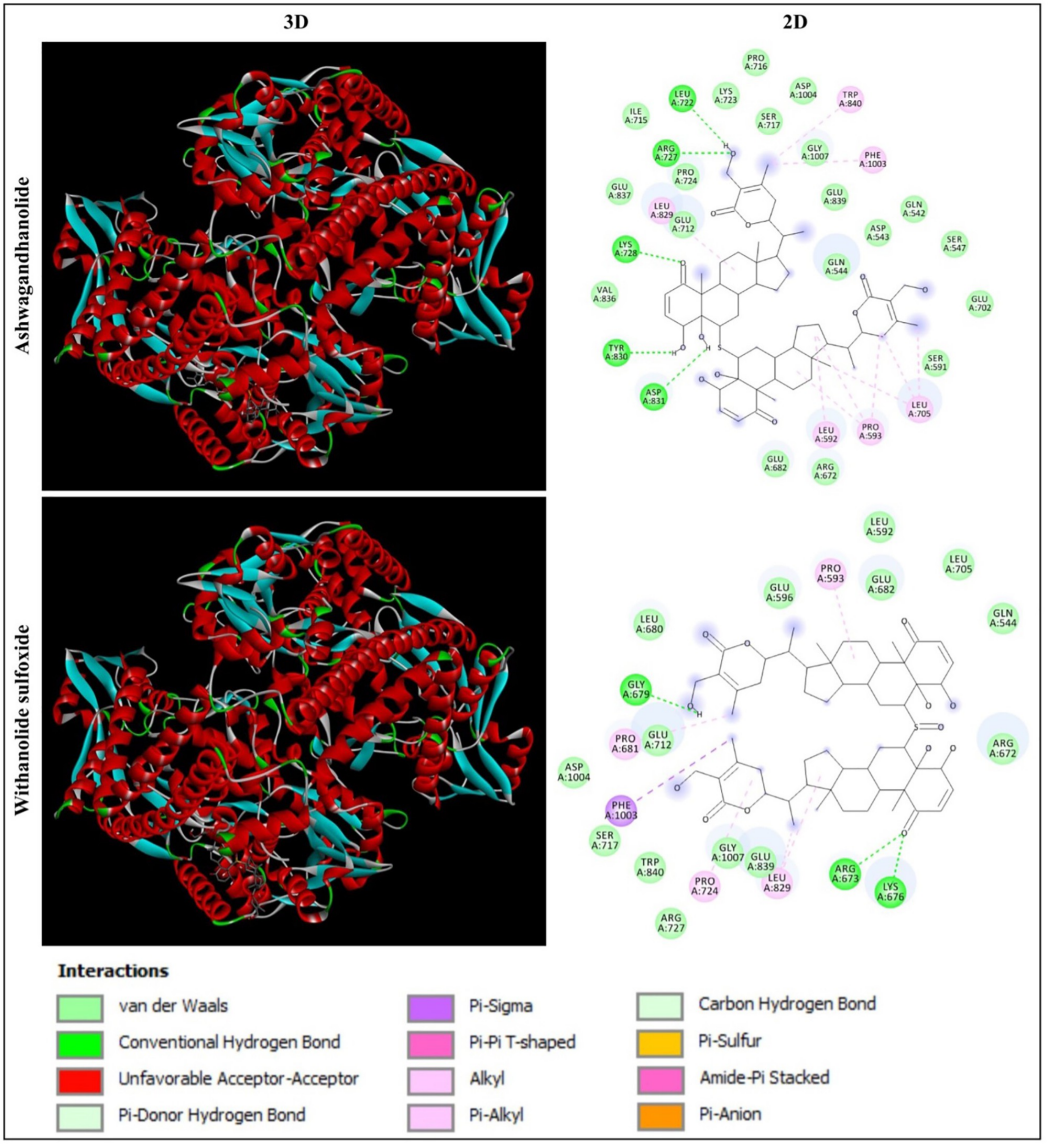
Interaction of human TOP2A (PDB: 5GWK) with Ashwagandhanolide and Withanolide sulfoxide. Three-dimensional (3D) illustration shows the interaction of ligands with human TOP2A structure and two-dimensional (2D) diagram displays the interactions of the ligand with the specific amino acid residues in the active site of the protein.

**Fig 7:**
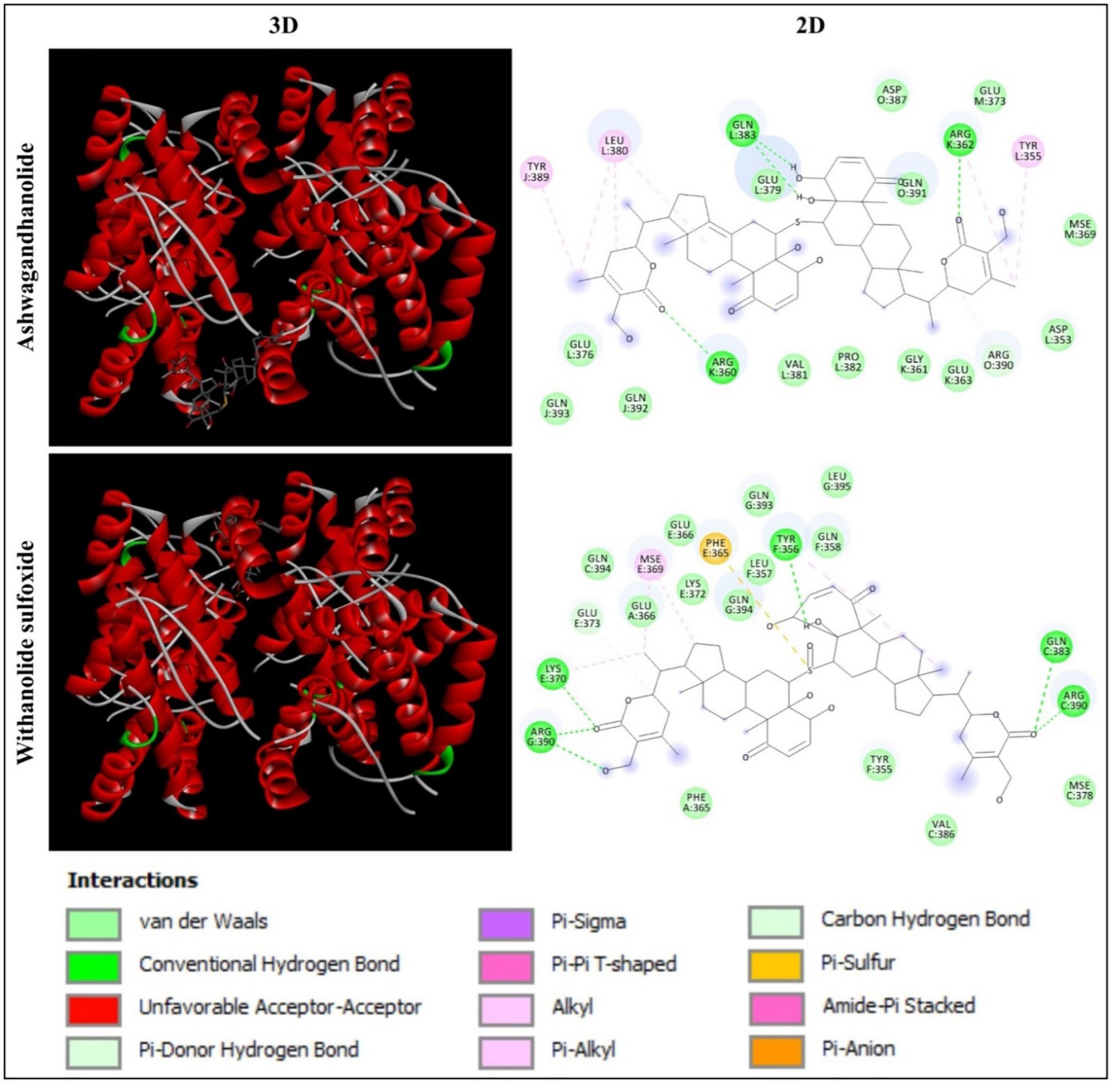
Interaction of human p73 tetramerization domain (PDB: 2WTT) with Ashwagandhanolide and Withanolide Sulfoxide. Three-dimensional (3D) illustration shows the interaction of ligands with human p73 tetramerization domain structure and two-dimensional (2D) diagram displays the interactions of the ligand with the specific amino acid residues in the active site of the protein.

Based on the molecular docking analysis of 44 phytochemicals from *W. somnifera*, Ashwagandhanolide and Withanolide sulfoxide, which exhibited the least binding energy scores of -9.6 to -9.7 kcal/mol, -11.6 to -12.7 kcal/mol, -10.8 to -12.1 kcal/mol and -11.0 to -11.2 kcal/mol have been identified as the most potent inhibitors against four different proteins (PDB: 6CBZ, 1FDW, 5GWK, and 2WTT, respectively) those expressed during breast cancer. The molecular docking analysis between these two compounds and target proteins showed the amino acid residues and interactions responsible for the stable protein–inhibitor complex formation. The results of the binding affinity (kcal/mol) of the compounds to the targeted multiple proteins are represented in Table 1. The interactions of Ashwagandhanolide and Withanolide sulfoxide with the active sites of ERα, 17β-HSD1, TOP2A and p73 tetramerization domain are shown in Figs 4–7, respectively.

**Table 1:** Molecular docking results of phytochemicals isolated from *W. somnifera* against four different proteins expressed during breast cancer.

SI No.	Compound Name	Binding Energy (Kcal/mol)			
		6CBZ	1FDW	5GWK	2WTT
1	Withanoside IV	-9.4	-10.0	-9.8	-10.3
2	Withanoside VI	-9.1	-9.5	-9.7	-11.0
3	Withanoside X	-9.0	-10.1	-9.6	-10.0
4	Withanoside D	-6.8	-8.4	-8.6	-7.5
5	Withanoside E	-7.0	-8.3	-8.4	-7.6
6	2,3-Dihydrowithanone-3β-O-sulfate	-8.7	-9.1	-9.0	-8.8
7	Coagulin Q	-8.6	-9.5	-9.0	-10.1
8	Physagulin D	-8.8	-9.7	-10.0	-10.0
9	2–3-Dihydro-3β-hydroxywhitanone	-8.5	-10.0	-9.3	-9.6
10	Viscosalactoneβ	-9.1	-9.8	-9.5	-8.5
11	27-Hydroxywithanolide A	-9.5	-9.9	-9.6	-9.4
12	6α,7α-Epoxy-3β,5, 20β-trihydroxy-1-oxowitha-24-enolide	-9.0	-10.0	-10.1	-10.3
13	(20S,22R)-3α,6α-Epoxy-4β,5β,27-trihydroxy-1-oxowitha-24-enolide	-8.9	-9.3	-10.3	-9.2
14	(20S,22R)-4β,5β,6α,27-Tetrahydroxy-1-oxowitha-2,24-dienolide	-8.5	-8.6	-9.2	-9.2
15	Dihydrowithanolide D	-10.0	-10.0	-9.5	-9.8
16	23,24-Dihydrowhitaferin A	-8.7	-8.9	-9.9	-8.7
17	Withanoside V	-9.5	-10.4	-10.1	-10.2
18	Withanolide A	-9.6	-10.3	-10.6	-10.7
19	Withaferin A	-9.3	-9.4	-9.8	-8.6
20	Withanone	-9.3	-9.7	-9.3	-9.5
21	Withanolide D	-9.3	-10.2	-10.1	-9.1
22	27-Hydroxywithanolide B	-9.3	-9.1	-9.4	-8.8
23	5,7α-Epoxy-6α,20α,20α-dihydroxy-1-oxowitha-2,24-dienolide	-8.5	-9.8	-10.1	-9.1
24	Isowithanone	-8.9	-9.8	-9.8	-9.8
25	Withanolide H	-9.2	-9.1	-9.3	-8.5
26	Withanolide K	-8.1	-9.8	-9.2	-9.4
27	Withanolide J	-9.3	-9.6	-9.1	-9.1
28	Δ^3^-Isowithanoliode F	-8.2	-8.6	-9.5	-8.9
29	Withanolide F	-8.5	-9.4	-9.2	-9.9
30	Withacoagulin G	-8.6	-9.8	-9.9	-9.2
31	Withacoagulin I	-8.6	-9.2	-9.6	-9.4
32	Withanolide S	-8.9	-9.5	-8.6	-9.1
33	Ixocarpalactone A	-8.7	-10.1	-9.8	-8.9
34	(20R,22R,24S,25R)-5β,6β-epoxy-4β,20β-dihydroxy-3β-methoxy-1-oxowithanolide	-9.4	-9.2	-9.3	-8.9
35	Ashwagandhanolide	-9.7	-12.7	-12.1	-11.0
36	Withanolide sulfoxide	-9.6	-11.6	-10.8	-11.2
37	Withanolide B	-9.5	-9.5	-9.4	-9.6
38	Withanolide G	-9.0	-9.7	-10.1	-10.0
39	Withasomidienone	-8.1	-9.6	-9.0	-8.9
40	Withacoagin	-9.9	-9.7	-9.8	-9.1
41	5β,6β-Epoxy-4β-hydroxy-1-oxowitha-2,24-dienolide	-8.3	-8.9	-9.6	-9.6
42	Withacoagulin E	-9.0	-9.7	-9.7	-10.1
43	Withacoagulin F	-9.1	-10.1	-9.9	-9.3
44	Withanolide I	-8.8	-9.4	-9.5	-9.5

#### Analysis of the interaction of ERα ligand-binding domain with Ashwagandhanolide and Withanolide sulfoxide

Estrogen receptor-α (ERα) is a ligand-inducible transcription factor in breast cancer that regulates the expression of specific DNA sequences in hormone response elements (HREs) responsible for important body functions [56, 57]. It was believed to increase the cell proliferation and metastasis capabilities in breast cancer cells by binding directly to the proximal promoter region of the oncogenes. The detection of ERα is considered as one of the main prognostic markers in breast cancer. Its status is crucial for breast cancer patients’ clinical diagnosis, management, and treatment decision-making process [57]. Therefore, inhibiting the ERα transcriptional activity in breast cancer cells would be a promising strategy for treating ER-positive breast cancer. Tamoxifen is the most widely used ERα modulator for breast cancer treatment, which has some main drawbacks, including the cause of uterine cancer, strokes, and pulmonary embolism.

In the present study, the molecular docking analysis of ERα (PDB: 6CBZ) with Ashwagandhanolide and Withanolide sulfoxide revealed that Ashwagandhanolide was found to form two hydrogen bonds with the amino acid residues GLU423 and LEU549, whereas Withanolide sulfoxide established two hydrogen bonds with the amino acid residues LYS520 and LYS531 (Fig 4). Ali et al [16] have been emphasized that the binding capability (-8.27 kcal/mol) of Withaferin A from *W. somnifera* via amino acid residues GLU353, ARG394 and LEU387 with ERα (PDB: 3ERD) using *in silico* approaches and elucidated it as a future breast cancer inhibitors targeting ERα. In addition, two molecules such as Withanolide D and Withanolide Q from *W. somnifera*, which had the good binding interaction and stability with the protein (PDB: 6CHZ), were reported as potential antagonists of ERα using in *silico* approach, drug-likeness analysis, and ADMET analysis [17].

#### Analysis of the interaction of the human 17β-HSD1 mutant protein with Ashwagandhanolide and Withanolide sulfoxide

Human 17β-HSD1 is a human steroid-converting enzyme that catalyzes the final steps in activating estrogens (especially estradiol), which promote the proliferation of hormone-dependent diseases like breast cancer [58, 59]. Its expression positively correlates with the activation of estrone, estradiol levels, and breast cancer cell proliferation. The expression and activity of 17β-HSD1 are much higher in breast cancer than in normal breast tissue, and this increased expression has been postulated as a possible explanation for the elevated estradiol content in breast cancer [58]. It is also known to be involved in the reduction of dehydroepiandrosterone (DHEA) into 5-androstene-3β,17β-diol (A-diol) and dihydrotestosterone (DHT) into 5α-androstane-3β,17β-diol (3β-diol) [59]. A-diol has been reported as the major estrogen found after menopause, whereas 3β-diol was capable of inducing the activation and proliferation of ERα. Hence, inhibition of 17β-HSD1 activity is also a very promising approach for the treatment of breast cancer.

In the present study, the molecular docking analysis of human 17β-HSD1 (PDB: 1FDW) with Ashwagandhanolide and Withanolide Sulfoxide revealed that Ashwagandhanolide was found to establish the hydrogen bonds with the amino acid residues SER11, ARG37, ALA191 and GLN221, whereas Withanolide sulfoxide formed the hydrogen bonds with the amino acid residues GLY92, TYR155, ALA191 and GLN221 (Fig 5). Froufe et al [60] have suggested that the low molecular weight compounds (such as 4-O-caffeoylquinic, naringin and lycopene) present in wild mushrooms were found as the potential 17β-HSD1 (PDB: 1FDT) inhibitors when searching for anti-breast cancer activity using molecular docking.

#### Analysis of the interaction of human TOP2A with Ashwagandhanolide and Withanolide sulfoxide

The TOP2A is an important enzyme in DNA replication in humans, particularly in breast cancer. It is considered the molecular target for most successful clinically active anticancer drugs, i.e., topoisomerase II (topo II) inhibitors [61]. The expression of the TOP2A protein has been linked to high proliferation and aggressive tumor subtypes in breast cancer, and it seems to be independent of its amplification status [62]. Unfortunately, the transcriptional regulation of expression of TOP2A remains largely unknown in solid tumors. The sensitivity to topo II inhibitors mainly depends on the expression level of TOP2A in targeted cancer cells. The TOP2A status could have predictive value in treatment or selecting a chemotherapy drug for breast cancer patients. Therefore, suppressing the activity of TOP2A using the new potential inhibitors is crucial for the inhibition of DNA replication in breast cancer, thereby preventing the disease.

In the present study, the molecular docking analysis of human TOP2A (PDB: 5GWK) with Ashwagandhanolide and Withanolide Sulfoxide revealed that Ashwagandhanolide was found to form the hydrogen bonds with the amino acid residues LEU722, ARG727, LYS728, TYR830 and ASP831, whereas Withanolide Sulfoxide established the hydrogen bonds with the amino acid residues ARG673, LYS676 and GLY679 (Fig 6). Saleh et al [63] have indicated that the binding affinity of mitoxantrone and its halogenated drug were found -9.2 and -10.3 kcal/mol, respectively, against TOP2A (PDB: 4FM9) inhibition by molecular docking calculation, which might be helped to develop a new anticancer drug.

#### Analysis of the interaction of human p73 tetramerization domain with Ashwagandhanolide and Withanolide sulfoxide

The p73 is the recently identified p53 family member in the cancer world which is required for p53-dependent apoptosis [64]. The over-expression of p73 can activate the p53-responsive elements, and p73 activation has been involved in G1/S cell cycle arrest and apoptotic cell death promoted by aberrant cancer cell growth and certain types of DNA damage [65]. As a result, the p73 may be particularly useful in treating cancerous cells by promoting apoptotic cell death in cancer cells lacking functional p53. The p73 may also be most important for the improvement of cancer cell chemosensitivity. The functional activation domains such as the tetramerization, DNA-binding, and N-terminal activation domains are essential for the p73 activity in transactivation and efficient growth inhibition [66]. As a result, decreasing the function of p73 as a pharmaceutical target for cancer therapy is critical for breast cancer prevention.

In the present study, the molecular docking analysis of the human p73 tetramerization domain (PDB: 2WTT) with Ashwagandhanolide and Withanolide Sulfoxide revealed that Ashwagandhanolide was found to establish the hydrogen bonds with the amino acid residues ARG360, ARG362 and GLN383, whereas Withanolide Sulfoxide formed the hydrogen bonds with the amino acid residues TYR356, LYS370, GLN383 and ARG390 (Fig 7). Sibuh et al [2] have revealed that the molecular docking interaction of the human p73 tetramerization domain (PDB: 2WTT) with 4-hydroxy benzaldehyde thiosemicarbazone (4-HBTSc) via two hydrogen bonding formed with leucine.

With these results obtained, all four proteins and potential ligand molecule (Ashwagandhanolide) complexes were further submitted to the MD simulation to evaluate the prevalence of the interactions predicted by the molecular docking studies.

### Molecular Dynamics (MD) simulation studies

**Fig 8:**
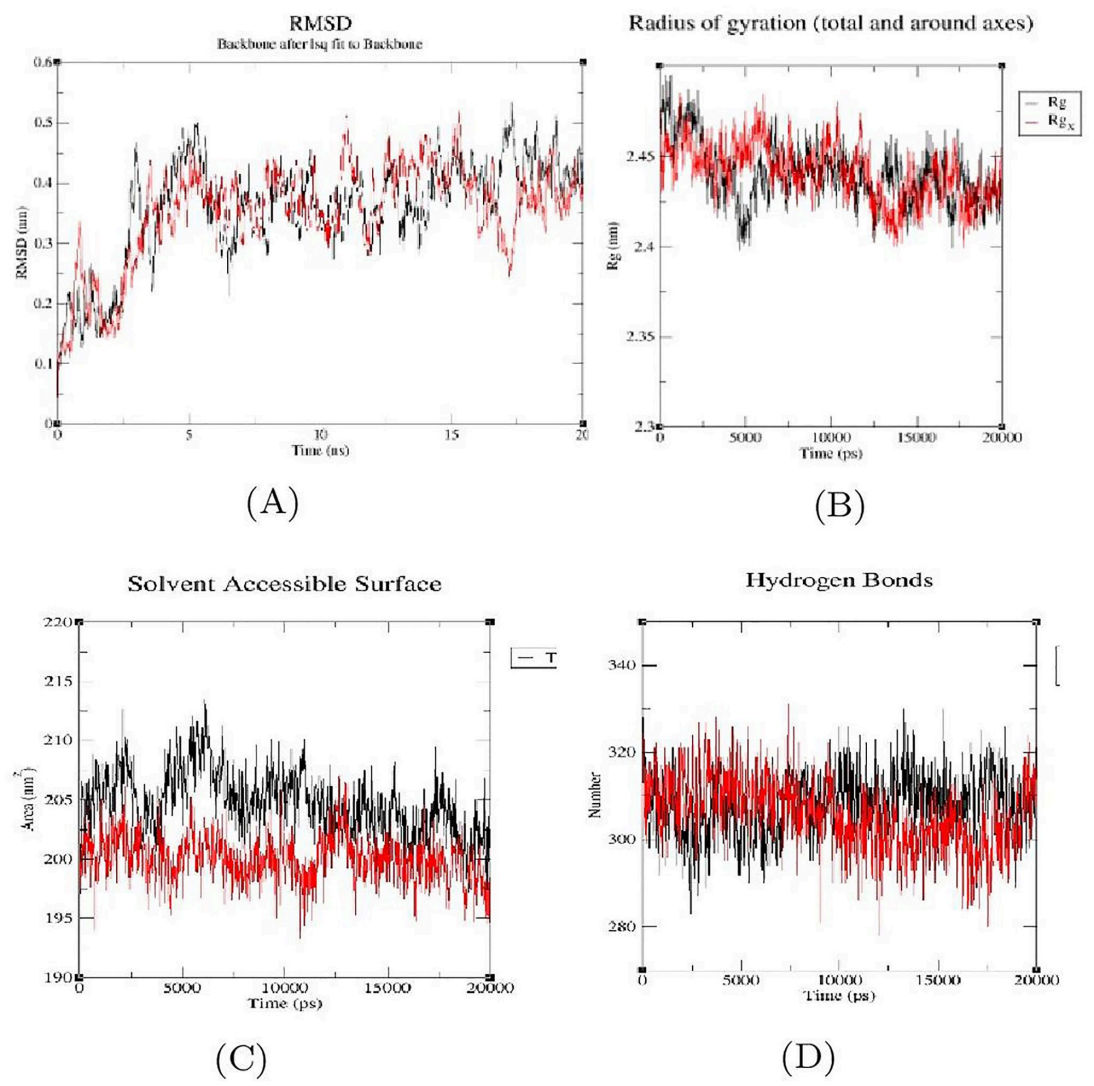
Analysis of RMSD (A), Rg (B), SASA (C) and Hydrogen (D) graphs of TOP2A (PDB: 5GWK) in complex with Ashwagandhanolide (Back color indicating protein alone and Red indicating protein-ligand complex).

The protein-ligand interaction with the lowest binding energy (i.e., high binding affinity) was chosen for further testing their complex stability under simulated conditions using MD simulation. The root means square deviation (RMSD), the radius of gyration (Rg), solvent accessible surface area (SASA), number of hydrogen bonds maintained during the simulation time, and variation in protein and their complexes were all examined in the simulation study. The simulation was run with the native protein alone and in complex with the ligand (Ashwagandhanolide) for 50 ns. The RMSD plot of all four proteins alone (Fig 8A) revealed that the proteins reached equilibrium around 13–20 ns and remained stable with minimal deviation in the 0.06–0.3 nm RMSD range, whereas the RMSD plot of proteins complexed with Ashwagandhanolide revealed that the proteins reached equilibrium at 0–11 ns and 0–7 nm (Fig 8A). When the proteins were in complex with Ashwagandhanolide, their structural flexibility was preserved. Ashwagandhanolide bound to the proteins attained equilibrium after the initial fluctuations.

The Rg is determined using root mean square distances and the central axis of rotation. Throughout the simulation, the Rg plot (Fig 8B) accounted for the capability, shape, and folding of the entire trajectory at every step. The Rg values of the four proteins complexed with Ashwagandhanolide followed a similar trend, with deviations ranging from 1.9 to 4.1 nm. The area around the hydrophobic core formed between protein-ligand complexes was evaluated with the SASA (Fig 8C. The SASA plot indicated that there were observed consistent SASA values. Over the period of simulation, the interaction with H-bonds that occurs during the molecular docking investigation was examined. All intermolecular H-bonds between proteins and Ashwagandhanolide were solely considered during the analysis and plotted accordingly. The plot showed that the number of H-bonds formed during MD simulation runs was consistent with the molecular docking study and only a few bonds were broken and repaired simultaneously.

### Prediction of ADMET properties of ligands

The ability of a substance to migrate through the intestinal epithelial barrier impacts its rate of movement and degree of human absorption, which directly affects its bioavailability. The molecular weight, H-bond donors, H-bond acceptors, log P, log Kp, logpMDCK, oral absorption, and log BB are based on the Lipinski Rule of Five which describes the molecular properties of drugs that are vital for their pharmacokinetics in the human body [67]. The chemical structural properties of Ashwagandhanolide are presented in Table 2. Ashwagandhanolide has several excellent chemical structural properties to interact with amino acid residues. It has maximum proton donors and acceptors, which are mainly crucial for forming hydrogen bonds to interact with amino acids. Ashwagandhanolide has the maximum LogP value (6.3768), which suggests its hydrophobic nature. All the ADMET properties of Ashwagandhanolide are depicted in Table 3. The rate of movement and degree of human absorption of any compound used as an oral drug is determined by its migration through the intestinal epithelial barrier, directly impacting its bioavailability. High Caco-2 permeability would result in anticipated values ≥ 0.90 in the computational model. Ashwagandhanolide has a positive Caco-2 value (0.38), which indicates its moderate human intestinal permeability attribute.

**Table 2:** Chemical structural properties of Ashwagandhanolide.

Descriptor	Value
Molecular Weight (g/mol)	975.295
LogP	6.3768
#Rotatable Bonds	8
#Acceptors	13
#Donors	6
Surface Area (Å^2^)	411.756

**Table 3:** Predicted ADMET properties of Ashwagandhanolide.

Descriptor	Model Name	Value
**Absorption**	Water solubility (log mol/L)	-3.159
	Caco2 permeability (log Papp in 10^−6^ cm/s	0.38
	Intestinal absorption (human) (% Absorbed	63.819
	Skin Permeability	-2.735
	P-glycoprotein substrate	Yes
	P-glycoprotein I inhibitor	Yes
	P-glycoprotein II inhibitor	Yes
**Distribution**	VDss (human) (log L/kg)	-1.652
	Fraction unbound (human) (Fu)	0.249
	BBB permeability	-1.006
	CNC permeability (log PS)	-2.859
**Metabolism**	CYP2D6 substrate	No
	CYP3A4 substrate	Yes
	CYP1A2 inhibitor	No
	CYP2C19 inhibitor	No
	CYP2C9 inhibitor	No
	CYP2D6 inhibitor	No
	CYP3A4 inhibitor	No
**Excretion**	Total Clearance (log ml/min/kg)	-0.679
	Renal OCT2 substrate	No
**Toxicity**	AMES toxicity	No
	Max. tolerated dose (human) (log mg/kg/day)	-0.058
	hERG I inhibitor	No
	hERG II inhibitor	No
	Oral Rat Acute Toxicity (LD50) (mol/kg)	3.704
	Oral Rat Chronic Toxicity (LOAEL) (log mg/kg_bw/day)	1.49
	Hepatotoxicity	No
	Skin Sensitization	No
	*T. pyriformis* (log μg/L)	0.285
	Minnow toxicity (log mM)	2.486

Note: Caco-2—Colon cancer cell line; Papp—apparent permeability coefficient; Kp—skin permeability constant; VDss—volume of distribution at steady state; Fu—fraction unbound; BBB—blood-brain-barrier; CBS—central nervous system; PS—permeability-surface area; CYP—Cytochrome P450; AMES—assay of the compounds ability to induce mutations in DNA; hERG—human ether-a-go-ago related gene; LD—lethal dose; LOAEL—lowest observed adverse effect level; *T. pyriformis—Tetrahymena pyriformis*.

The drug’s absorption from an orally administered solution is predicted by the intestinal absorption (human) value. This value for Ashwagandhanolide is greater than 60%, indicating good absorption. This compound also has a predicted skin permeability value (-2.735) ≤ -2.5 log Kp, indicating poor skin permeability. Because P-glycoprotein is a component of the ATP-binding cassette (ABC) transporter, the value ‘yes’ for P-glycoprotein substrate indicates the chemical can be carried across the cell membrane via ABC transporter. Here, Ashwagandhanolide is predicted to be transported across the ABC transporter. The volume of distribution (VDss) refers to the total number of drugs distributed uniformly in the blood. A VDss value of less than -0.15 log VDss indicates a low VDss value, while a value of more than 0.45 log VDss indicates a significantly high VDss value. The compound Ashwagandhanolide used in the present study has a relatively low VDss value (-1.652).

The BBB permeability determines whether or not a substance may enter the brain. Because the compounds with log BB values ≥ 0.3 are thought to pass through BBB, Ashwagandhanolide did not easily cross the BBB. Furthermore, the BBB permeability isn’t required for targeting S-protein, therefore it’s not a desirable attribute for the current goal. Besides, Ashwagandhanolide is believed to have the permeability of the CNS because its log PS value (-2.859) is less than -2.0, which is good for only the drugs used to treat the nervous system diseases. The metabolism predictions indicated that Ashwagandhanolide has little effect on cytochrome function, with no inhibition of CYP1A2, CYP2C19, CYP2C9, CYP2D6 and CYP3A4. The renal excretion of Ashwagandhanolide showed the lowest value (-0.679 log ml/min/kg) of total clearance. Ashwagandhanolide does not show AMES toxicity suggesting that this compound does not possess mutagenicity. This was not expected to cause any hepatotoxicity and skin sensitization. The cardiac potassium channels hERG I and II are encoded by human ether-a-go-go-related gene (hERG), and their blockage can result in QT (i.e., the peaks of the heart ECG) syndrome, which impairs the heart’s repolarization after a heartbeat. Ashwagandhanolide was not found to show such inhibition. Recently, Patel et al [68] have suggested that Ashwagandhanolide from *W. somnifera* was found to be one of the most potent antiviral agents which can be used as an orally administered drug based on their ADMET characteristics and recommended it for further studies. It is noteworthy that the binding affinity of the withanolides with human serum albumin plays a decisive role in pharmacodynamics and pharmacokinetics thereby indicating its biological importance. In addition, it is well noted that the ADMET studies are conduct to predict how the structural features of the phytoconstituents interact with the amino acids of the targeted proteins [68].

### Conceptual DFT studies

The estimated values for the Global Reactivity Descriptors (including the Nucleophilicity N) [34–40] for the Ashwagandhanolide moelecular system acquired utilizing the in-house CDFT tool software are displayed in Table 4:

**Table 4:** Global Reactivity Descriptors of the Ashwagandhanolide molecular system.

*χ*	*η*	*ω*	S	N	*ω* ^−^	*ω* ^+^	Δ*ω*±
4.0217	3.8986	2.0744	0.2565	2.8215	6.4032	2.3815	8.7848

Note: *χ*—Electronegativity; *η*—Global Hardness; *ω*—Electrophilicity; S—Global Softness; N—Nucleophilicity; *ω*^−^—Electrodonating Power; *ω*^+^—Electroaccepting Power; Δ*ω*±—Net Electrophilicity. All the descriptors are expressed in eV, with the exception of S, which is expressed in eV^−1^.

The electronegativity *χ* and global hardness *η* are absolute chemical reactivity parameters for which no experimental analogue exists. Indeed, the observed vertical ionization energy (I) and vertical electron affinity (A) can be used to approximate them, but these values are unknown for the molecular system under investigation. The electrophilicity index is a balance between an electrophile’s proclivity for acquiring more electron density and its reluctance to exchanging electron density with its surroundings [69]. A classification of organic compounds as strong, moderate, or marginal electrophiles, that is an electrophilicity scale, was established by considering a group of Diels-Alder reactions and the electrophiles involved in them [70–72], with larger than 1.5 eV for the first case, between 0.8 and 1.5 eV for the second case, and smaller than 0.8 eV for the final case [70–72], On the basis of Table 4, the Ashwgandhanolide molecule may be classified as a moderate electrophile. Domingo and colleagues [69, 71, 73–75] presented a Nucleophilicity index N based on the HOMO energy calculated using the KS technique with an arbitrary shift of the origin, using the molecule of tetracyanoethylene (TCE) as a reference. They were able to classify organic molecules as strong nucleophiles with N > 3.0 eV, moderate nucleophiles with 2.0 < N < 3.0 eV, and marginal nucleophiles with N < 2.0 eV after analyzing a series of common nucleophilic species involved in polar organic processes. By re-examining Table 4, it is clear that the three molecular systems may be also classified as a moderate nucleophile.

**Fig 9:**
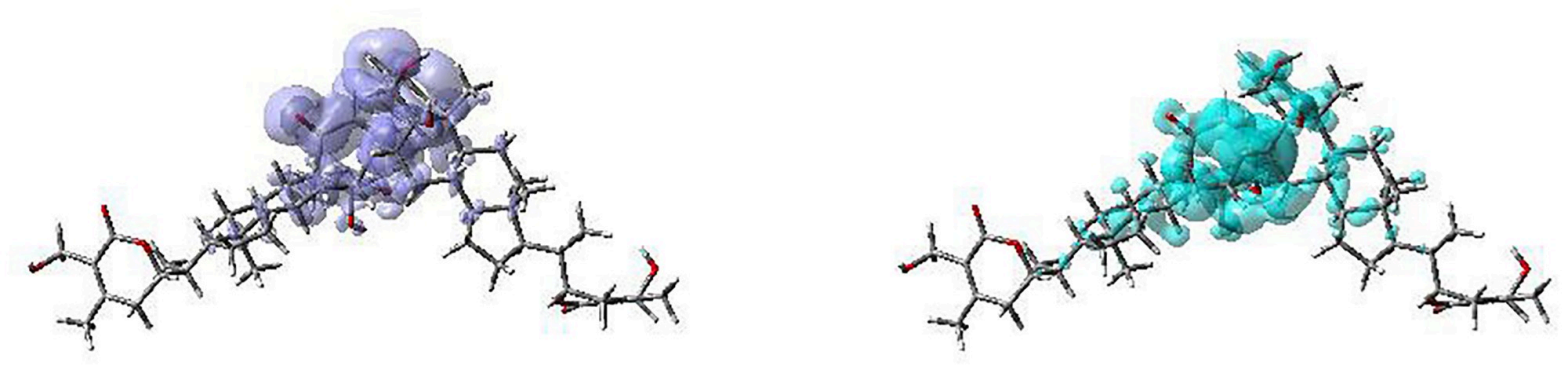
Graphical representation of the dual descriptor DD of Ashwangandhanolide. Top: DD > 0, Bottom: DD < 0.

The Dual Descriptor Δ*f*(**r**) or DD [38, 76–80], has been shown to describe unambiguously nucleophilic and electrophilic sites within a molecule [80]. A graphical representation of the DD for the Ashwagandhanolide molecular system is displayed in Fig 9 showing the zones where DD > 0 and DD < 0:

## Conclusion

*Withania somnifera* is a medicinal plant that possesses a treasure of active metabolites of biological importance including the anticancer compounds (Withanolide A, Withanone, Withanoside IV, Withanoside VI, Withaferin A and Withaferin D). The present study on molecular docking and simulation identifies a potential anticancer compound apart from the well-known anticancer compounds which are Ashwagandhanolide and Withanolide sulfoxide. During the docking analysis, it was noted that out of the 44 compounds, Ashwagandhanolide and Withanolide sulfoxide had the potentiality to inhibit the four different proteins (viz., ERα, 17β-HSD1, TOP2A and p73 tetramerization domain) responsible for breast cancer. The MD simulation studies authenticated that Ashwagandhanolide inhibited all the four proteins by forming the dead-end complex. Further, the ADMET analysis of Ashwagandhanolide inferred that the compound was hydrophobic with moderate intestinal permeability, good intestinal absorption, poor skin permeability and relatively low VDss value (-1.652) indicating that the compound did not easily cross the blood-brain barrier (BBB). The Conceptual DFT study performed through the analysis of the chemical ractivity descriptors revealed the favorable characteristics of this compound for its consideration as a good therapeutic drug. The results of the study indicate that Ashwagandhanolide may be a potential breast cancer drug candidate which needs attention.

## Supporting information

S1 File 3D X-Ray crystallographic structures of the selected breast cancer target protein receptors and extended and detailed version of the materials and methodology employed including additional references. (PDF)
