# Supplementary material for: Phytoconstituents of Withania somnifera unveiled Ashwagandhanolide as a potential drug targeting breast cancer: Investigations through computational, molecular docking and conceptual DFT studies
Source: PLoS One. 2022 Oct 6;17(10):e0275432. doi: 10.1371/journal.pone.0275432 (PMC9536605; doi:10.1371/journal.pone.0275432)
Supplement: S1 File — (PDF) [file pone.0275432.s001.pdf]

SUPPLEMENTARY DATA

Details of methodology

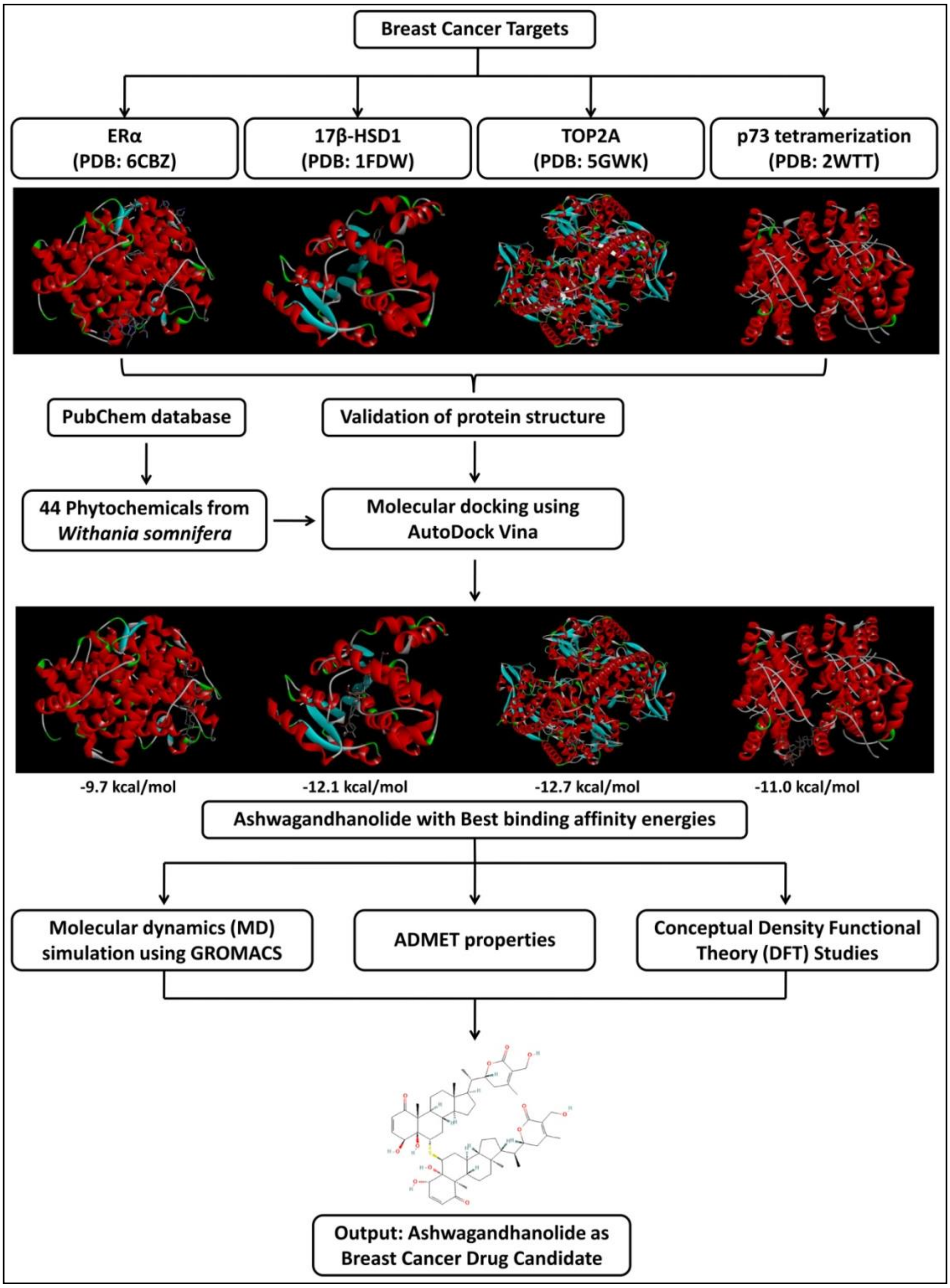

Preparation of ligands

The three-dimensional (3D) chemical structures of 44 phytochemicals from *Withania somnifera* were considered as ligand molecules for the present study [Trivedi et al., 2017]. The chemical structures of all the selected phytochemicals which were available in the PubChem database (https://pubchem.ncbi.nlm.nih.gov/) were retrieved in Structured Data Format (.sdf) and that were unavailable were drawn using the Chemaxon's chemical drawing tool (MarvinSketch version 18.30) (https://chemaxon.com/products/marvin/). The ligand preparation was performed using the OpenBabel version 2.3.2 and exported in the protein data bank (pdb) format [Pires et al., 2015] and were used for molecular docking analysis.

Preparation of target protein structures

Based on the literature, four main therapeutic targets for the breast cancer *viz*., estrogen receptor alpha (ERα) ligand-binding domain Y537S mutant in complex with estradiol and GRIP peptide, human 17-beta-hydroxysteroid-dehydrogenase type 1 (17β-HSD1) mutant H221Q complexed with estradiol, human topoisomerase II alpha (TOP2A) in complex with DNA and etoposide, and human p73 tetramerization domain were selected in the present study [Sibuh et al., 2021]. The 3D X-Ray crystallographic structures of the selected breast cancer target protein receptors (ERα, 17β-HSD1, TOP2A and p73 tetramerization domain) were retrieved as pdb format from the Protein Data Bank database (https://www.rcsb.org/) (Suppl. Table 1). The protein structures were processed by removing the co-crystallized ligand and water molecules attached to them to avoid unwanted molecular interactions with the target receptors during virtual screening exercise using the Discovery Studio software [Pradeep et al., 2021]. The energy minimization, reconstruction of missing atoms and stereo-chemical quality checks of the protein receptors were performed by using the same software.

Supplementary Table 1. 3D X-Ray crystallographic structures of the selected breast cancer target protein receptors

Protein	PDB ID	Resolution	HTTP Link
ERα	6CBZ	1.65 Å	https://www.rcsb.org/structure/6CBZ
17β-HSD1	1FDW	2.70 Å	https://www.rcsb.org/structure/1FDW
TOP2A	5GWK	3.15 Å	https://www.rcsb.org/structure/5GWK
p73 tetramerization domain	2WTT	2.30 Å	https://www.rcsb.org/structure/2WTT

Validation of protein structure

The quality of protein structure was further validated using the Ramachandran plot by using PROCHECK via PDBsum database (http://www.ebi.ac.uk/thornton-srv/databases/pdbsum/Generate.html). The plot enables the visualization of highly preferred, preferred, and disallowed phi (φ) and psi (ψ) angles of each amino acid present in the protein [Prasad et al., 2021]. In addition, the protein structure was also checked with the Protein Structure Analysis (ProSA) web tool for protein model quality assessment. The selected protein that showed Z-score within the range of their respective native proteins (for determining the high-quality) validates their quality.

Molecular docking study

The molecular docking analysis of ligands against the target protein receptors were completed by using Autodock Vina which implicated in PyRx [Uppar et al., 2021] and the resulting binding affinities were expressed in kcal/ mol. The grid box (i.e., binding pocket) for the XYZ coordinates was at 60 Å × 60 Å × 60 Å in case of 6CBZ and 1FDW, while it was at 120 Å × 120 Å ×120 Å in 5GWK and 2WTT. The whole target receptors were enclosed within the dimensions of grid box with exhaustiveness of 100 poses. The protein coordinates were saved in pdbqt file format, and the docking parameters were set as default. The conformation with the least binding energy was considered as the best docking pose. The molecular interactions of protein-ligand complexes produced were visualized with the help of Discovery Studio software [Vasantha Kumar et al. 2021].

Molecular dynamics (MD) simulations

The molecular dynamics (MD) simulations of the complexes of all the four protein receptors with Ashwagandhanolide were carried out to understand the stability of identified ligand molecule with the protein receptors. The MD simulations were performed using the GROMACS 5.1.4 software with the GROMOS96 43A1 force-field. The ligand topology files were created with the help of the PRODRG server. The prepared protein-ligand complex was then solvated in a cubic box of edge length 10 nm around the central SPC water molecule [Prasad et al., 2020; Dharmashekara et al., 2021]. The adequate number of NaCl counter ions and co-ions was added to maintain the electroneutrality of system. The systems were minimized to remove the short contacts and atoms overlaps. The cut-off radius of 0.9 nm was applied for both van der Waals and Coulombic interactions. The Particle Ewald Mesh method was used to describe the long-range electrostatic interactions. The equilibration was done in

two steps. At first step, the coordinates of protein-ligand complex were restrained at their respective positions, and the solvent and ions were allowed to relax in the canonical (NVT) ensemble. In the second step, the restraint weights from the protein-ligand complexes were gradually reduced, and the whole system was equilibrated in the isothermal-isobaric (NPT) ensemble. Using the LINCS algorithm, all bonds including hydrogen atoms were restrained. The temperature of system was controlled at 300 K using the Berendsen thermostat while the pressure was maintained at 1 bar using the Parrinello-Rahman barostat. The long production simulations were initiated with configurations collected from the previous equilibration step. All the systems were simulated for 20 ns in the NPT ensemble, and trajectory frames were saved at every two ps interval [Chadha et al., 2015].

Assessment of Absorption, Distribution, Metabolism, Excretion and Toxicity (ADMET) properties

The ADMET was used to predict the absorption, distribution, metabolism and toxicity properties of the selected compounds. These properties are very important during drug development processes for any phytochemicals. The online pkCSM platform was used to investigate the ADMET properties of the potential ligand molecule [Pires et al., 2015].

The following were the predicted ADMET properties:

Absorption: Water solubility (log mol/L)
Caco2 permeability (log Papp in 10-6 cm/s)
Human intestinal absorption (% Absorbed)
Skin Permeability (log Kp)
P-glycoprotein substrate
P-glycoprotein I inhibitor
P-glycoprotein II inhibitor

Distribution: Human VDss (log L/kg)
Human fraction unbound (Fu)
BBB permeability (log BB)
CNS permeability (log PS)

Metabolism: CYP2D6 substrate
CYP3A4 substrate
CYP1A2 inhibitor
CYP2C19 inhibitor
CYP2C9 inhibitor

CYP2D6 inhibitor

CYP3A4 inhibitor

Excretion: Total Clearance (log ml/min/kg)

Renal OCT2 substrate

Toxicity: AMES toxicity

Human Max. tolerated dose (log mg/kg/day)

hERG I inhibitor

hERG II inhibitor

Oral Rat Acute Toxicity (LD50) (mol/kg)

Oral Rat Chronic Toxicity (LOAEL) (log mg/kg_bw/day)

Hepatotoxicity

Skin Sensitization

Tetrahymena pyriformis toxicity (log ug/L)

Minnow toxicity (log mM)

References:

Trivedi, M.K.; Panda, P.; Sethi, K.K.; Jana, S. Metabolite profiling in Withaniasomnifera roots hydroalcoholic extract using LC/MS, GC/MS and NMR spectroscopy. Chem. Biodivers. 2017, 14, e1600280. https://doi.org/10.1002/cbdv.201600280

Pires, D.E.V.; Blundell, T.L.; Ascher, D.B. pkCSM: predicting small-molecule pharmacokinetic and toxicity properties using graph-based signatures. J. Med. Chem. 2015, 58, 4066-4072. https://doi.org/10.1021/acs.jmedchem.5b00104

Pradeep, S.; Jain, A.S.;Dharmashekara, C.; Prasad, S.K.;Akshatha, N.;Pruthvish, R.;Amachawadi, R.G.; Srinivasa, C.; Syed, A.;Elgorban, A.M.; Al Kheraif, A.A.; Ortega-Castro, J.; Frau, J.; Flores-Holguín, N.;Shivamallu, C.; Kollur, S.P and Glossman-Mitnik, D. Synthesis, Computational Pharmacokinetics Report, Conceptual DFT-Based Calculations and Anti-Acetylcholinesterase Activity of Hydroxyapatite Nanoparticles Derived From Acorus Calamus Plant Extract. *Front. Chem.* ***2021***, 9,741037. doi: 10.3389/fchem.2021.741037

Sibuh, B.Z.; Khanna, S.; Taneja, P.; Sarkar, P.; Taneja, N.K. Molecular docking, synthesis and anticancer activity of thiosemicarbazone derivatives against MCF-7 human breast cancer cell line. *Life Sci.* **2021,** *273,* 119305. https://doi.org/10.1016/j.lfs.2021.119305

Prasad, S.K.; Pradeep, S.;Shimavallu, C.; Kollur, S.P.; Syed, A.;Marraiki, N.;Egbuna, C.;Gaman M-A.;Kosakowska, O.; Cho, W.C.; Patrick-Iwuanyanwu, K.C.; Ortega Castro, J.; Frau, J.; Flores-Holguín, N and Glossman-Mitnik, D. Evaluation of Annona muricata Acetogenins as Potential Anti-SARS-CoV-2 Agents Through Computational Approaches. *Front. Chem.* ***2021***, 8,624716. doi: 10.3389/fchem.2020.624716

Uppar V, Chandrashekharappa S, Shivamallu C, P Sushma, Kollur SP, Ortega-Castro J, Frau J, Flores-Holguín N, Basarikatti AI, Chougala M, Mohan M M, Banuprakash G, Jayadev, Venugopala KN, Nandeshwarappa BP, Veerapur R, Al-Kheraif AA, Elgorban AM, Syed A, Mudnakudu-Nagaraju KK, Padmashali B, Glossman-Mitnik D. Investigation of Antifungal Properties of Synthetic Dimethyl-4-Bromo-1-(Substituted Benzoyl) Pyrrolo[1,2-a] Quinoline-2,3-Dicarboxylates Analogues: Molecular Docking Studies and Conceptual DFT-Based Chemical Reactivity Descriptors and Pharmacokinetics Evaluation. *Molecules*. ***2021***, 26(9),2722.

Vasantha Kumar, RamithRamu, Prithvi S Shirahatti, V B Chandana Kumari, P Sushma, Subhankar P Mandal and Shashank M Patil. α-Glucosidase, α-Amylase Inhibition, Kinetics and Docking Studies of Novel (2-Chloro-6-(trifluoromethyl)benzyloxy)arylidene) Based Rhodanine and Rhodanine Acetic Acid Derivatives.*Chemistry select*. ***2021***, 6(36), 9637-44.

Dharmashekara C, Pradeep S, Prasad S K, Jain A S, Syed A, Prasad K S, Patil S S, Beelagi MS Srinivasa C and Shivamallu C. Virtual screening of potential phyto-candidates as therapeutic leads against SARS-CoV-2 infection. *Environmental Challenges*, ***2021***, 4, 100136. doi:10.1016/j.envc.2021.100136

Prasad A, Shruthi G, Sushma P, Jain AS, Chandan D, Prasad MNN, et al. Helicobacter pylori Infection: A Bioinformatic Approach. International Journal of Pharmaceutical Sciences and Research. 2020;11(11):5469{5483. doi:10.13040/ijpsr.0975-8232.11(11).5469-83.

Chadha N, Tiwari AK, Kumar V, Milton MD, Mishra AK. In Silico Thermodynamics Stability Change Analysis Involved in BH4 Responsive Mutations in Phenylalanine hydroxylase: QM/MM and MD Simulations Analysis. Journal of Biomolecular Structure and Dynamics. 2015;33(3):573-583. doi:10.1080/07391102.2014.897258.
